# Heterogeneity of Tumor Vasculature and Antiangiogenic Intervention: Insights from MR Angiography and DCE-MRI

**DOI:** 10.1371/journal.pone.0086583

**Published:** 2014-01-23

**Authors:** Wenlian Zhu, Yoshinori Kato, Dmitri Artemov

**Affiliations:** 1 Division of Cancer Imaging Research, The Russell H. Morgan Department of Radiology and Radiological Science, The Johns Hopkins University School of Medicine, Baltimore, Maryland, United States of America; 2 Department of Oncology, The Sidney Kimmel Comprehensive Cancer Center, The Johns Hopkins University School of Medicine, Baltimore, Maryland, United States of America; European Institute of Oncology, Italy

## Abstract

**Purpose:**

Solid tumor vasculature is highly heterogeneous, which presents challenges to antiangiogenic intervention as well as the evaluation of its therapeutic efficacy. The aim of this study is to evaluate the spatial tumor vascular changes due to bevacizumab/paclitaxel therapy using a combination approach of MR angiography and DCE-MRI method.

**Experimental Design:**

Tumor vasculature of MCF-7 breast tumor mouse xenografts was studied by a combination of MR angiography and DCE-MRI with albumin-Gd-DTPA. Tumor macroscopic vasculature was extracted from the early enhanced images. Tumor microvascular parameters were obtained from the pharmacokinetic modeling of the DCE-MRI data. A spatial analysis of the microvascular parameters based on the macroscopic vasculature was used to evaluate the changes of the heterogeneous vasculature induced by a 12 day bevacizumab/paclitaxel treatment in mice bearing MCF-7 breast tumor.

**Results:**

Macroscopic vessels that feed the tumors were not affected by the bevacizumab/paclitaxel combination therapy. A higher portion of the tumors was within close proximity of these macroscopic vessels after the treatment, concomitant with tumor growth retardation. There was a significant decrease in microvascular permeability and vascular volume in the tumor regions near these vessels.

**Conclusion:**

Bevacizumab/paclitaxel combination therapy did not block the blood supply to the MCF-7 breast tumor. Such finding is consistent with the modest survival benefits of adding bevacizumab to current treatment regimens for some types of cancers.

## Introduction

Intratumoral vascular heterogeneity is an important feature of the solid tumors [1], [2] and needs to be considered when the therapeutic response to a targeted antiangiogenic regimen is evaluated. Contrast-enhanced MR angiography (MRA) is an ideal tool with which to investigate the heterogeneity of the tumor vasculature due to its ability to visualize the static macroscopic vessels of the tumor with high spatial resolution and strong contrast [3]. Combining MRA with a dynamic contrast-enhanced MRI (DCE-MRI) study of the microvasculature, it is possible to obtain a more comprehensive picture of the tumor vascular function [4]. Nevertheless, the current common practice is to use DCE-MRI results only to evaluate the treatment response to single or combination antiangiogenic treatment. Although the American College of Radiology Breast Imaging Reporting and Data System Atlas (BI-RADS® Atlas) lexicon did utilize the internal enhancement pattern as well as the dynamic curve characteristics in the diagnosis of malignancy [5], therapeutic efficacy of targeted antiangiogenic treatments is conventionally measured by changes in statistical values representing microvascular permeability, averaged over the entire tumor or large tumor regions like hot spots only [6]. Such a global analysis typically results in poor correlation with clinical outcomes [6]. One of the reasons could be that any significant local and regional vascular changes due to the therapy may be masked and lost in the averaging process. Indeed, it is time to rethink the approach of using a single number to quantitatively measure the efficacy of a targeted antiangiogenic regimen without considering intratumoral heterogeneity.

Another obstacle in using averaged values, such as mean or median in therapeutic assessment, is that tumor microvascular parameters often have an abnormal skewed distribution over the entire tumor due to the spatial heterogeneity [7]. Direct comparison of the longitudinal mean or median of the same tumor, or of different tumors, is not meaningful, as these values cannot represent the complexity of non-normal distribution. There have been some exploratory efforts to quantify tumor vascular heterogeneity in order to characterize the tumor vascular network more accurately and to detect the differential regional microenvironment changes in the tumor in response to treatments [7], [8], [9]. Initial vascular heterogeneity quantification attempts were either region-based, in which the tumor was divided into multiple concentric bands of less spatial variability [10] or histogram-based [11]. Other approaches, such as principle component analysis, texture analysis, and Rényi fractal dimension and geometrical property analysis, were proposed as well [12]. The region-based method is mostly useful in animal models of solid tumors where a “rim enhancement pattern” is commonly observed, so that the tumor can be segmented into a poorly enhancing core and a strongly enhancing periphery or rim in an “onion-peeling” manner [8]. While histograms constructed from the voxel-by-voxel DCE-MRI parametric maps adequately depict the heterogeneity within the tumor, quantitative analysis of such histograms in response to treatment remains arbitrary and challenging [13]. As such, neither region- nor histogram-based methods have addressed the intrinsic spatial complexity of the intratumoral heterogeneity and thus, have not produced reliable quantitative biomarkers of prognostic value.

The mismatch between tumor growth and vascular supply leads to hypoxia and the up-regulation of multiple cytokines, such as vascular endothelial growth factor A (VEGF-A), which increases microvascular permeability and plays a dominant role in angiogenesis [14]. Significant hypoxia tends to occur in low-perfusion areas [15]. One study showed that mean interstitial pH and mean oxygen pressure decreased at an increased distance from a blood vessel [16]. The largest proportion of hypoxia was found at distances beyond 100 µm from perfused vessels in a human glioma mouse model [17]. To address the unique connection between vascular perfusion and microvascular permeability within the tumor, we proposed using a spatial analysis of the tumor microvascular parameters based on the macroscopic vascular architecture detected by contrast-enhanced MRI with a macromolecular contrast agent (MMCA), albumin-Gd-DTPA. Tumor *macrovasculature* composed of these macroscopic vessels was extracted using early time-point DCE-MRI images and tumor microvascular parameters were analyzed voxel-by-voxel based on the distance from the voxel to the nearest macroscopic blood vessels. Vascular changes were evaluated in a human breast tumor MCF-7 mouse model treated with bevacizumab/paclitaxel combination therapy. Although a similar approach had been adapted in the immunohistochemical analysis of the spatial relationship between hypoxia and the perfused vascular network [17], to our best knowledge this is the first application of this method to the analysis of the *in vivo* functional microvascular parameters. The present study provided clear imaging evidence that the tumor blood supply was not blocked by the bevacizumab/paclitaxel combination treatment in the MCF-7 breast tumor mouse model, and corroborated the modest survival benefits of adding bevacizumab to current treatment regimens for some types of cancers.

## Materials and Methods

### Human Breast Cancer Xenografts in Mice

The human breast MCF-7 mouse xenografts were established as described previously [18]. Briefly, 3×10^6^ MCF-7 cells (in 0.05 ml of Hanks’ balanced salt solution) were inoculated into the thoracic mammary fat pad of the SCID mice 48 hours after the implantation of one-half of a 17β-estradiol pellet in the back of the mouse.

### Ethics Statement

All animal experiments in this study were approved and performed in accordance with the Guidelines for Animal Experimentation of the Johns Hopkins University School of Medicine and every effort was made to minimize suffering.

### Treatment and Scanning Schedule

Combination therapy with bevacizumab and paclitaxel was initiated after tumors reached approximately 150 mm^3^ in volume. MCF-7 breast tumor xenograft-bearing mice were treated with three doses of bevacizumab and paclitaxel by intraperitoneal injection, each at a dose of 10 mg/kg every four days (n = 7). DCE-MRI scanning was performed prior to the treatment and on day 12 after the initiation of the treatment. Mice in the control group were treated with equal volumes of saline and were subjected to the same scanning protocol (n = 6) [18]. Tumor volumes were determined from the length of the longest axis and the width of the vertical axis, measured with a slide caliper, as described previously [19]. Overall, 14 mice were scanned prior to the treatment, 7 and 6 mice were scanned at day 12 for the combination treatment and the control group, respectively [18].

### MRI Data Acquisition and Analysis

MRI data acquisition and analysis were performed as described earlier [18]. Briefly, 3D DCE-MRI images were acquired using a highly T1-weighted, saturation recovery, short delay gradient echo sequence. The total acquisition time for a 3D image set was about six minutes, with a field of view of 12×12×10 mm, a matrix size of 128×64×48, and six averages. A baseline image was acquired before the intravenous administration of albumin-Gd-DTPA at 0.16 mmol (Gd)/kg, and dynamic contrast-enhanced images were acquired for 30 min after the injection. Raw data were processed with an in-house IDL program (Exelis) to a final image size of 128×128×128. The contrast concentration in voxels was calculated directly from the equilibrium magnetization M_0_ map, pre and post-contrast signal amplitudes, and the blood relaxation rate as described [18]. Images were visualized using the ImageJ (National Institutes of Health) and Amira (Visage Imaging) packages.

### Modeling CA Pharmacokinetics from DCE-MRI Data

Under the above experimental conditions, the tumor vascular volume (*VV*) and vascular permeability-surface area product (*PS*) for each voxel can be derived directly from the intersect and slope of the linear regression of the enhanced intensity versus elapsed time after administration of albumin-Gd-DTPA, respectively, as described previously [20], [21]. Only positive intercepts and slopes were retained as physically relevant *VV* and *PS* values [18].

### Segmentation of Tumor Macrovasculature and Heterogeneity Analysis

A binary map of the macroscopic tumor blood vasculature was generated semi-automatically from the initial enhanced images acquired immediately after the administration of albumin-Gd-DTPA based on a combination of threshold, cluster size, and boundary identification process. The segmented continuous voxels above a set threshold formed the binary macrovasculature map. A 3D voxel-by-voxel distance map was subsequently created by calculating the distance from each voxel to the nearest macroscopic blood vessel. The tumor microvascular parameters, *VV* and *PS,* were then analyzed based on the distance from the nearest blood vessel. Statistical analysis was performed using the non-parametric Wilcoxon signed-rank test. The tumor macrovascular vessel volume ratio was calculated, using the total voxel counts of segmented vessels and total tumor volumes determined slice-by-slice from the T1-weighted images.

## Results

### Segmentation of the Tumor Macrovasculature

**Figure 1:**
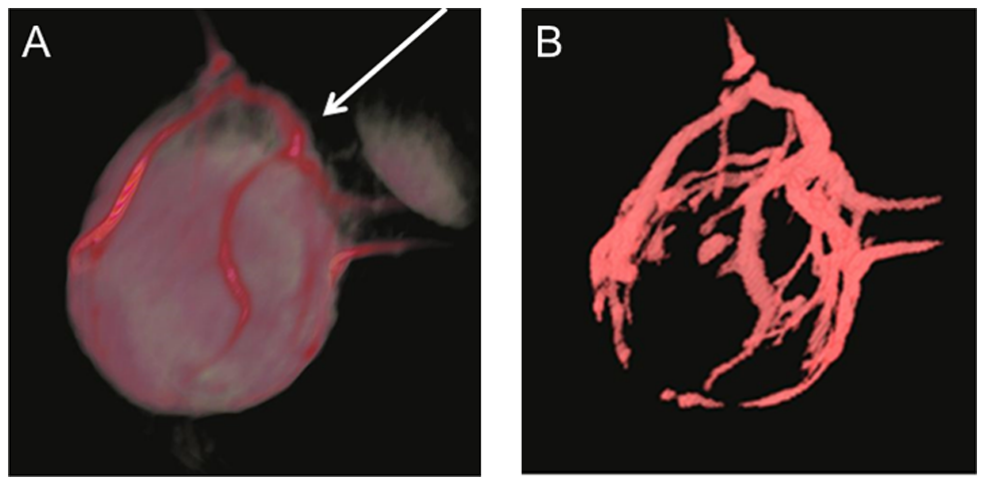
Segmentation of the tumor macrovasculature. A: A representative 3D rendering of the images of MCF-7 tumor orthotopically grown in a female SCID mouse acquired immediately after the injection of albumin-Gd-DTPA. B: corresponding segmented binary macrovasculature map. The arrow indicates where the tumor was attached to the mouse body.

The vasculature of MCF-7 tumors was, indeed, quite heterogeneous. Tumor angiograms were obtained from the early dynamic enhanced images, acquired within six minutes after the administration of albumin-Gd-DTPA, when the leakage of the MMCA to the tumor interstitium was minimal. The macroscopic vessel/background contrast threshold ratio varied slightly between the mice in our investigation, possibly due to the individual fluctuations in the albumin-Gd-DTPA injection dose, and mouse cardiac and kidney functions. Nevertheless, the strong contrast between the macroscopic vessels and the remainder of the tumor allowed the unambiguous segmentation of the tumor macrovasculature using a semi-automatic IDL program, adapting a threshold in the range of the upper 15–20 percentiles of the enhancement. An example of a 3D rendering of the aforementioned enhanced image is shown in Fig. 1, on the left, while the corresponding binarized segmented macrovasculature map is shown on the right. Noticeably, strongly enhanced macroscopic feeding vessels were detected extending from the body flank into the tumor periphery in all mice in both the bevacizumab and paclitaxel combination treatment and saline control groups in various time points. The images in Fig. 1 were acquired prior to the treatments.

### Distance to the Nearest Macroscopic Vessel

**Figure 2:**
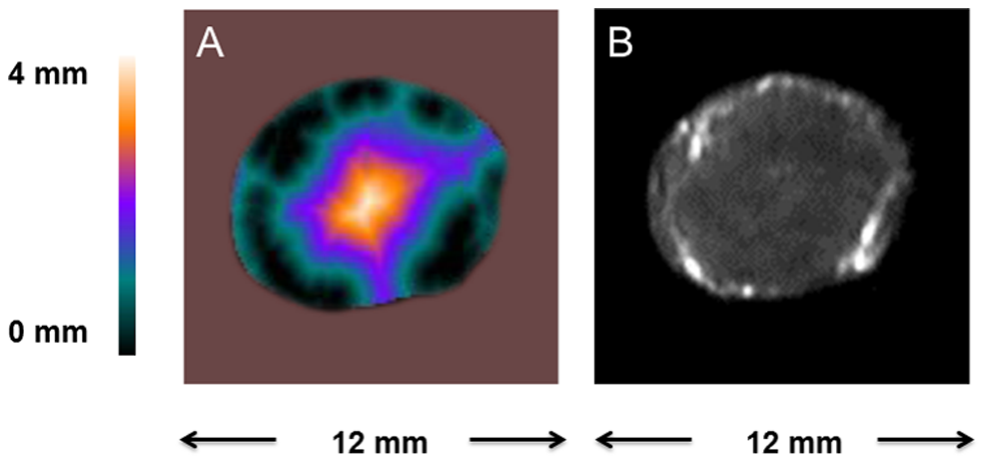
Generation of the distance map. A: A representative central slice from a 3D tumor distance map that depicts the distance of each pixel to the nearest segmented macroscopic vessel. B: the corresponding early enhanced image.

A central slice from the 3D distance map that depicts the distance from each pixel to the nearest macroscopic vessel within the tumor, is shown in Fig. 2, as well as the corresponding early enhanced image. Fig. 2 shows that a sizable portion of the tumor peripheral area is in close vicinity to the macroscopic vessels, while the tumor core area is generally poorly enhanced and farther away from these vessels. Similar distance maps were observed in the pre- and post-treatment mice that received either saline or the bevacizumab and paclitaxel combination treatment. The images in Fig. 2 were acquired prior to the treatments.

### Distribution of the Tumor Microvascular Parameters

**Figure 3:**
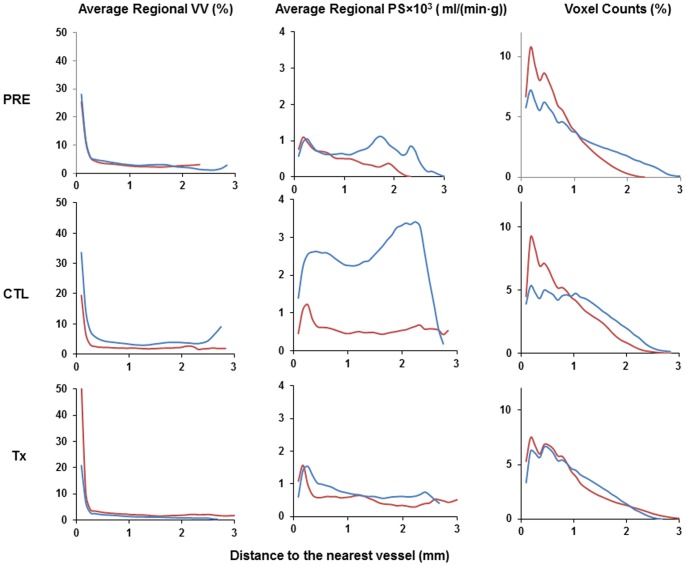
Representative distribution of average tumor VV and PS as a function of the distance to the nearest segmented vessel. Results for two mice, shown in red and blue lines, from the pre-treatment (PRE), day 12 of saline control (CTL), and day 12 of bevacizumab/paclitaxel combination (Tx) groups are shown.

Charts that plot representative average regional tumor *VV* and *PS,* as well as the corresponding voxel counts, stratified by the distance to the nearest enhanced vessel are shown in Fig. 3. Not surprisingly, areas in the vicinity of the tumor macroscopic vessels displayed the highest average vascular volume. Tumor areas close to these vessels also generally showed higher average *PS* values. Average *VV* decreased rapidly with increasing distance to the vessels and leveled off at around 250 µm. Treatment with bevacizumab/paclitaxel combination therapy did not have a significant impact on the overall distribution pattern of the tumor microvascular parameters, Fig. 3.

### Vascular Changes Induced by the Bevacizumab/Paclitaxel Combination Therapy

**Figure 4:**
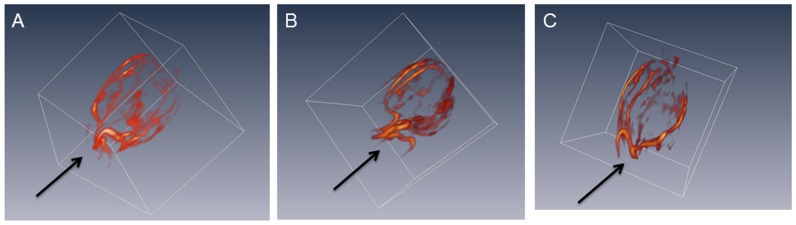
Tumor macrovasculature extracted from the 3D T1-weighted images acquired immediately after the administration of albumin-Gd-DTPA. A: pre-treatment, B: day 12 of saline control, C: day 12 of bevacizumab/paclitaxel combination treatment. The arrows indicate where the tumor was attached to the mouse body.

Bevacizumab/paclitaxel combination therapy appeared to have induced barely any changes in the tumor macrovascular network, as evidenced by the tumor angiograms from different treatment groups shown in Fig. 4. The average macrovascular volume fraction was 4.4% (±1%) of the total tumor volume prior to the start of the treatment, and was 4.2% (±1%) and 4.8% (±1%) at the end of the treatment for the saline control and the bevacizumab/paclitaxel group, respectively. Overall, 18% (±5%) of the entire tumor volume was within 250 µm of an enhancing vessel at the initiation of the treatment, with an average tumor volume of 170 mm^3^. Twelve days later, at the end of the treatment, 23% (±3%) of the total tumor volume remained within 250 µm of the nearest enhancing vessel in mice that received bevacizumab/paclitaxel, with an average tumor size of 160 mm^3^. In control mice that received saline, the average tumor volume was 300 mm^3^ and the fraction of tumor within a 250 µm range of an enhancing vessel was 17% (±5%).

**Figure 5:**
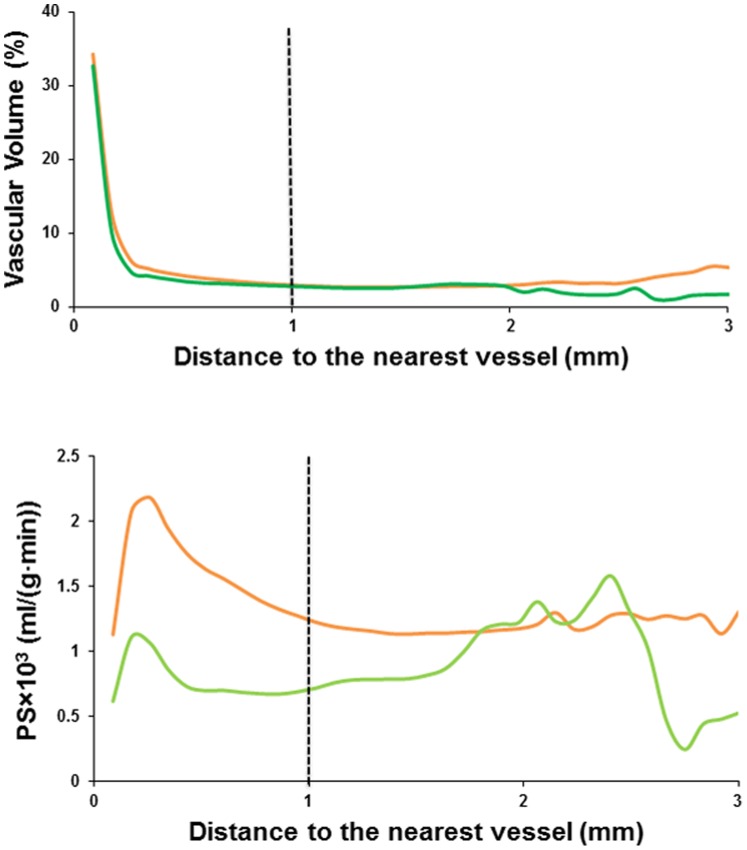
Distribution of group average VV and PS values of the control (orange) and the treated mice (green) relative to the distance to the segmented nearest vessel. The control group was the combination of the pre-treatment and day 12 of saline treated mice.

A trend of decreasing permeability *PS*, averaged over the entire tumor, was observed in the mice received the bevacizumab/paclitaxel combination treatment, in comparison to the *PS* in the pre-treated or the saline controlled mice [18]. However, a consistent decrease in both *VV* and *P*S in the treated mice was apparent when the stratified average values were compared base on the distance to the nearest vessel, as shown in Fig. 5. The overall albumin-Gd-DTPA enhancement in the tumor core was very low [18]. As such, the determination of *VV* and *PS* in these regions, with the distance to the nearest vessel above approximately 1 mm, was mainly dominant by the signal intensity fluctuation due to the noise. When these core regions were excluded from the analysis, the decreases in *VV* and *PS* in the group of treated *vs.* controlled mice were significant in the tumor area within 1 mm to the nearest enhancing vessel with p values less than 2⋅10^−5^, respectively.

## Discussion

DCE-MRI with an MMCA, albumin-Gd-DTPA, enabled the noninvasive acquisition of high-resolution 3D tumor angiograms (macrovasculature) as well as determination of the functional microvascular tumor parameters. Although it has been suggested that tumor blood supply and therapeutic intervention that targets the tumor vasculature may be more accurately assessed with a combination of DCE-MRI and MR angiography [4], past studies focused primarily on the microvascular changes only [6]. Our study is one of the few attempts to provide a more complete *in vivo* mapping of the tumor vasculature that includes larger vessels and small leaky capillaries, each an indispensable part of a functional vascular network. While MR angiography can detect only the macroscopic enhancing blood vessels that are close to or larger than the spatial MRI resolution, on the order of 100 µm in our study, DCE-MRI can reveal the presence and the permeability of micro-capillaries that are well below the imaging resolution by the kinetic analysis of the contrast concentration [22].

In this study, we detected a general trend of decreased *VV* and *PS* with increasing distance from large enhancing blood vessels. It has been reported that the diffusion limit for oxygen and nutrients is around 100 to 200 µm from the blood vessels [23], [24], and tumors could not grow to more than 1–2 mm^3^ without neovascularization [2], [25], [26]. Consistent with this diffusion limit, our results showed that *VV*, a measurement of the total macro- and microvascular volume, drastically decreased at increasing distance from the vessels and leveled off around 250 µm.

Inadequate vascular supply and the subsequently induced hypoxia are the driving force of angiogenesis and increased vascular permeability [2]. While the general trend of decreased *PS* observed in the current study appears in contradiction with the possible severe hypoxia at increasing distance from macrovasculature [15], [16], [17], the product of vascular permeability and vascular surface area, *PS,* is also related to the vascular volume. Low *PS* values observed might be partially due to the low vascular surface area in the poorly perfused regions with low vascular volume and might not have reflected the possible high vascular permeability in these regions.

A significant decrease in average *VV* and *PS* values were detected in the vicinity of the macroscopic vessels within the tumor after the bevacizumab/paclitaxel combination treatment. However, as demonstrated by Fig. 4, the tumor macrovasculature is largely unresponsive to the bevacizumab/paclitaxel combination treatment. This is not surprising, as the feeder vessels and draining veins are considered to have originated from remodeling and enlargement of the preexisting arteries and veins [27]. Such blood vessels are mature and are less angiogenically active [27]. These vessels can survive antiangiogenic therapy, replenish the blood and nutrient supply to the remaining tumor cells, and maintain an avenue for tumor growth and expansion. An increased portion of the MCF-7 tumor was within the close distance of a macroscopic blood vessel after the bevacizumab/paclitaxel combination treatment, possibly due the tumor growth retardation. Previous study also shows that treatment with antiangiogenic agent SU5416 results in larger vessel perimeter and vessel area in a rat mammary cancer model [28]. The persistence of such macroscopic vessels in the established tumors diminishes the significance of any microvascular changes by a targeted antiangiogenic regimen. Indeed, bevacizumab treatment was initiated within 24 hours of the tumor cell inoculation in one of the first reports that demonstrated the potent efficacy of this VEGF antibody in a mouse model of human cancer, prior to the establishment of any tumor vasculature [29]. Clinical trials showed that the addition of bevacizumab to neoadjuvant chemotherapy for the early stage breast cancer patients resulted in a modest but significant increase in the rate of pathological complete response [30], [31]. However, bevacizumab is currently indicated for the first- or second-line treatment of patients with advanced stage metastatic carcinoma [32]. It is possible that the indifference of the mature macroscopic vessels in the well-established tumors towards bevacizumab contributes to the marginal improvement in overall survival rates when it is added to a treatment regimen for late stage metastatic breast cancer patients [33], [34], [35], [36]. The inhibition of angiogenesis may only improve progression-free survival rates temporarily in such situation [33], [34], [35], [36].

The tumor angiogram observed here showed that MCF-7 tumors are supplied by large vessels on the MRI resolution scale, which closely paralleled the normal organs such as muscles, kidney and brain. Areas outside the major vessels remained less enhanced, yet these organs can carry out their normal functions while any vascular occlusion can have serious consequences. Similarly, permanent occlusion of feeding arteries and draining veins in solid mouse tumors by vascular targeted photodynamic therapy leads to tumor necrosis and eradication within 24–48 hours [37]. Our combined angiogram and DCE-MRI results reaffirm the need to identify new tumor blood vessel type-specific targets for a more comprehensive therapeutic intervention at different stages of tumor developments [27], [38]. Our approach also stresses the importance of developing robust prognostic imaging biomarkers to design and evaluate treatment options based on tumor characteristics.
